# Short-Term Learning Effects of a Cardiopulmonary Resuscitation Program with Focus on the Relationship between Learning Effect and Trainees’ Perceived Competence

**DOI:** 10.3390/healthcare9050598

**Published:** 2021-05-18

**Authors:** Kazunori Akizuki, Hideki Koeda

**Affiliations:** Department of Physical Therapy, Kobe International University, Kobe 658-0032, Japan; koeda@kobe-kiu.ac.jp

**Keywords:** cardiopulmonary resuscitation, skill, training, intrinsic motivation, competence, self-efficacy

## Abstract

High-quality education and training are essential for effectively improving the quality of cardiopulmonary resuscitation (CPR); however, the relationship between the acquisition of motor skills and learners’ psychological characteristic has not been investigated fully. Therefore, we investigated the relationship between intrinsic motivation for training, self-efficacy for CPR, and CPR skill acquisition through training. Twenty health sciences undergraduate students participated in a 3-hour basic life support course. Their chest compression skills were assessed before and after the course. The main outcome of this study was the chest compression score, with changes in the score from pretest to posttest regarded as the short-term learning effects from training. The chest compression score was significantly higher after the course (median 53.5%, interquartile range [IQR] 39.8–83.0) than before the course (median 14.0%, IQR 0–43.3, *p* < 0.001). Furthermore, we found a significant correlation between perceived competence after the training and changes in the chest compression score from pretest to posttest (r = 0.483, *p* = 0.031), but other psychological indices did not correlate with changes in the chest compression score. A significant correlation was noted between trainees’ perceived competency and the short-term learning effects of CPR training. We suggest instructors focus on psychological components of training, including trainees’ perceived competence.

## 1. Introduction

High-quality cardiopulmonary resuscitation (CPR) improves survival rates and patient outcomes following out-of-hospital cardiac arrest (OHCA) [1,2,3,4]. Current guidelines recommend a chest compression depth of 50–60 mm, compression rate of 100–120 compressions/min, compression fraction of >60%, and 30 compressions interrupted by two rescue breaths [5]. When the optimal compression depth of 50–60 mm is applied, a higher survival rate with an improved neurological outcome is observed [6,7,8]. However, the risk of trauma increases when the depth of compression is greater than 60 mm [9]. Complete recoil of the chest wall during CPR generates negative intrathoracic pressure, and consequently, improves venous return and cardiac output. If the chest is not allowed to adequately recoil, then the intrathoracic pressure tends to increase. This then decreases both blood flow to the right side of the heart as well as coronary perfusion pressure, which cumulatively, reduce myocardial blood flow [10]. Idris et al. analyzed the relationship between chest compression and survival discharge rates and revealed that the survival discharge rate decreases as the chest compression rate increases beyond the appropriate range [11]. 

Education and training are essential to effectively improve the quality of CPR delivered to the patient. Therefore, there are numerous studies to identify better educational methods to improve basic life support (BLS) skill effectively and achieve long term retention. Kovács et al. investigated the influences of timing of testing on skill retention, and found that testing skills 3 months after training may be more effective than either testing immediately after training or not testing at all [12]. Moreover, real-time visual feedback devices [13,14,15], audio–visual feedback devices [16,17], and smartwatch feedback devices [18,19,20] have shown to improve the quality of CPR training. With the use of effective feedback devices and improvement in the quality of training, trainees are able to improve their skill and retain it longer. However, we believe that the effectiveness of CPR training may be influenced by a psychological component, such as trainees’ intrinsic motivation. Therefore, despite providing good training, it may not be effective unless trainees are personally motivated to acquire the skills. 

In the motor learning research domain, it has been reported that there is a deep relationship between the acquisition of motor skills and the psychological aspects of learners [21]. In particular, learners’ intrinsic motivation, self-efficacy, and perceived competency are affected by motor learning [22,23,24]. While self-efficacy is defined as situation-specific self-confidence [25], competence is considered to be a basic psychological need and an important source of human motivation [26,27]. These terms are clearly distinguished both conceptually and empirically [28]. Some previous studies reported that learners’ self-efficacy and CPR skills correlated significantly [29,30], but another study reported that self-efficacy was not predictive of skills [31]. Thus, a consistent view has not yet been obtained in this regard. With regard to intrinsic motivation, to the best of our knowledge, there are no reports investigating this relationship with the acquisition of CPR skills.

Therefore, we aimed to conduct a quantitative evaluation of the CPR skills of the trainees before and after the CPR training program. We also measured trainees’ intrinsic motivation for training and self-efficacy for CPR to clarify the relationship between the psychological aspects of trainees and CPR skill acquisition.

## 2. Materials and Methods

### 2.1. Study Design and Participants

We conducted a prospective, quasi-experimental pretest–posttest study at Kobe International University, Kobe, Japan. In this study, 20 health sciences undergraduate students (6 female, 14 male; mean age 18.6 ± 0.5 years) who participated in the BLS course conducted in September 2020 were enrolled. Six of the participants had already undergone BLS training before participating in this study (5.7 ± 2.4 months since the last training). No participants in the BLS course declined participation in the study.

The study protocol was approved by the Institutional Review Board of Kobe International University (approval no. G2020-156). Before the initiation of the study, we explained the details of the experiment to all potential participants. Subsequently, we obtained written informed consent from all participants.

### 2.2. Equipment and Measurement Outcome

We used two types of mannequins. One mannequin was used to assess trainees’ CPR skills (Resusci Anne QCPR, Laerdal Medical, Stavanger, Norway) and manage the measurement data by connecting it with a software program (Wireless Skill Reporter, Laerdal Medical, Stavanger, Norway). The other mannequin was a standard, BLS training mannequin (Resusci Anne, Laerdal Medical, Stavanger, Norway). In this study, we measured chest compression-related indices including the depth and rate of chest compressions. The chest compression score, which was calculated according to an algorithm developed by Laerdal Medical (Stavanger, Norway), was also used. Because the score decreases with deviations from ideal performance, a lower overall score indicates a larger deviation from the ideal performance.

We also measured intrinsic motivation for BLS training and self-efficacy for chest compressions in CPR. To measure participants’ intrinsic motivation, the Intrinsic Motivation Inventory (IMI) was used [32]. The validity of this scale for various motor tasks has been confirmed [24,33,34]. Similar to previous studies [33,34], a nine-item questionnaire consisting of interest/enjoyment, perceived competence, and effort/importance sub-scales of the IMI was adapted for use in the present study. Likert scale responses ranged from 1 = strongly disagree to 7 = strongly agree. Internal consistency testing using Cronbach’s-α statistic was found to be acceptable for interest/enjoyment (0.71), perceived competence (0.74), and effort/importance (0.66) subscales. Although the Cronbach’s-α coefficient of effort/importance was slightly below the generally accepted level of 0.7, the values of the coefficients obtained in this study were similar to those obtained in previous studies [33,34].

All participants were asked to answer a question on their self-efficacy in performing CPR skills (i.e., How confident are you in performing a resuscitation attempt?) [30]. The participants rated the question on a 10-point scale, from 1 = not at all to 10 = completely confident.

### 2.3. Study Procedures

**Figure 1:**
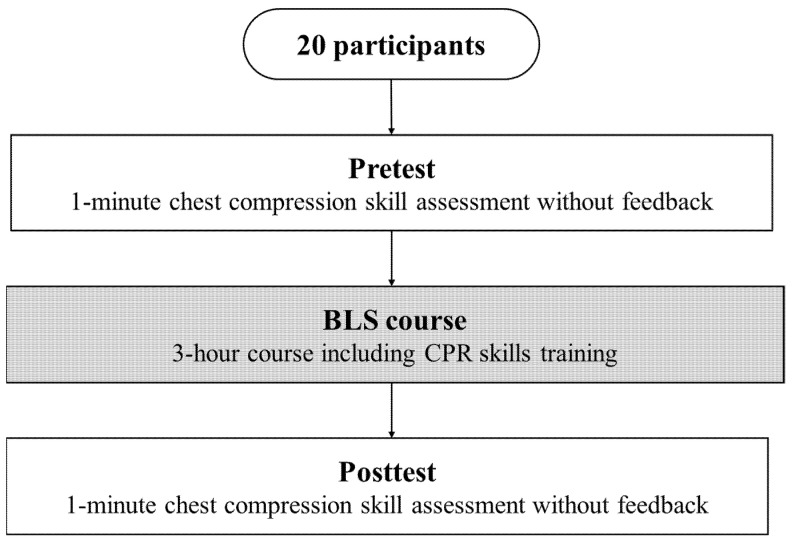
Experimental protocol. All 20 basic life support (BLS) course attendees underwent a pretest, the BLS course, and a posttest to measure the chest compression skills before and after a BLS course with cardiopulmonary resuscitation (CPR) skills training.

The protocol of this study is shown in Figure 1. Before the measurement, we conducted an orientation program for the participants, wherein information on the appropriate range and the number of chest compressions per minute as indicated in the Japan Resuscitation Council (JRC) Resuscitation Guidelines 2015 [35] was communicated in both written format and verbally. 

After the orientation, a pretest was conducted using a mannequin for assessing CPR skills. The pretest aimed to confirm the skill level of participants before attending the BLS training course. During pretest, participants were instructed to perform chest compressions for 1 minute. We chose a duration of 1 minute to minimize the deterioration of the quality of chest compressions due to fatigue [36]. Nishiyama et al. recommended that CPR providers change their roles every 1 minute to maintain the quality of chest compressions during chest compression-only CPR [36]. Fatigue is a factor that deteriorations performance during CPR and can make short-term learning effects difficult to detect. Therefore, we adopted 1 minute chest compression-only CPR for testing. Immediately after the pretest, the participants were asked to answer the IMI and self-efficacy measurements.

The 3-hours BLS course was conducted by licensed BLS instructors. In this course, the participants gained knowledge and psychomotor skills relative to BLS, including CPR and the use of an automated external defibrillator. When the participants gained hands-on practice, standard mannequins were used. The instructor:trainee:mannequin ratio was 1:3:1, and the maximum ratio was 1:4:1.

After the training course, the participants were required to answer the IMI and self-efficacy measurement again. Then, the participants performed the posttest with the same content as that in the pretest. The posttest aimed to confirm changes in chest compression skills.

### 2.4. Outcomes

The primary outcome of this study was the chest compression score. Because this index includes various aspects related to chest compression, the extent of change in this index from pretest to posttest was regarded as the effect of the BLS course.

The secondary outcomes were chest compression depth, percentage of compressions performed at the correct depth, percentage of compressions fully released, chest compression rate, percentage of compressions performed at the correct rate, IMI score, and self-efficacy score. The percentage of compressions performed at the correct depth and rate was calculated based on the adequate range described in the JRC Resuscitation Guidelines 2015 [35]. 

### 2.5. Statistical Analysis

The mean value and standard deviation were calculated for variables whose normality was confirmed and compared between pretest and posttest using paired t-tests. In contrast, for variables for which normality was not confirmed, the median and interquartile range (IQR, 25th–75th percentiles) were calculated and compared using the Wilcoxon signed-rank sum test. In addition, correlation analysis was performed to clarify the relationship between the extent of change in the chest compression score and the subscales of IMI or self-efficacy. 

We used Excel statistical software (BellCurve for Excel, Social Survey Research Information Co., Ltd., Tokyo, Japan) for all statistical analyses. In all analyses, a significance level of *p* < 0.05 was used.

## 3. Results

**Figure 2:**
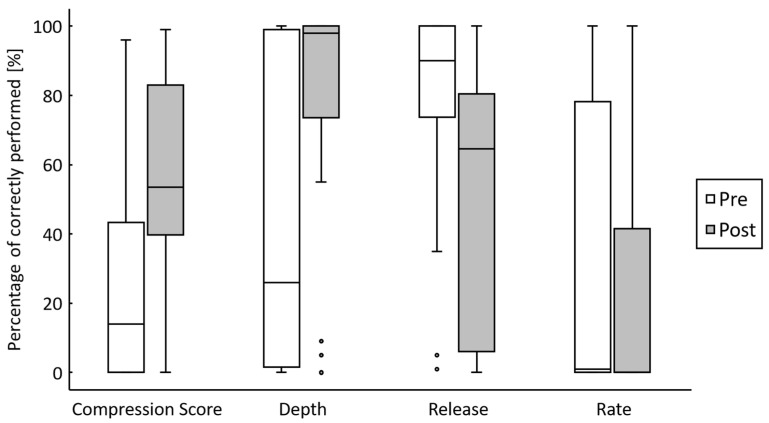
Comparisons of chest compression quality between pretest and posttest. The chest compression score is a comprehensive index reflecting various aspects related to chest compression. The depth and rate indicate the percentage of compressions, which were correctly performed against the appropriate range, as described in the guideline during the 1-minute chest compression test. The release indicates the percentage of compression, which were fully released. The box plots represent the interquartile range (IQR), the line inside represents the median, the whiskers represent 1.5 times the IQR, and the circles represent outliers.

The chest compression score after the course (median 53.5%, IQR 39.8–83.0) was significantly higher than that before the course (median 14.0%, IQR 0–43.3) (W = 11, Z = 3.380, *p* < 0.001, r = 0.756) (Figure 2). Furthermore, the median depth of chest compressions before the course was 45.0 mm (IQR 39.0–54.0), while after the course, it was 55.0 mm (IQR 50.8–57.0), which was significantly deeper (W = 0, Z = 3.724, *p* < 0.001, r = 0.833). The percentage of compressions performed at the correct depth improved significantly from before the course (median 26.0%, IQR 1.5–99.0) to after the course (median 98%, IQR 73.5–100.0) (W = 4, Z = 3.309, *p* < 0.001, r = 0.740). In contrast, while the percentage of compressions fully released was 90.0% (IQR 73.8–100.0) before the course, it decreased significantly to 64.5% (IQR 6.0–80.5) after the course (W = 19, Z = 3.058, *p* = 0.002, r = 0.684). The rate of chest compressions increased significantly from 130.5/min (IQR 105.5–136.0) before the course to 131.5/min (IQR 122.5–141.3) after the course (W = 40.5, Z = 2.193, *p* = 0.028, r = 0.490). The percentage of compressions at the correct rate was not significantly different after the course compared to that before the course (median 1.0, IQR 0–78.3 and median 0.0, IQR 0–41.5, respectively) (W = 37, Z = 0.594, *p* = 0.553, r = 0.133). 

Interest/enjoyment, which is a subscale of IMI, improved significantly from that before the course (median 5.0, IQR 4.3–5.4) to that after the course (median 5.8, IQR 5.3–6.8) (W = 1.5, Z = 3.763, *p* < 0.001, r = 0.841). The perceived competence was significantly higher after the course than that before the course (before: 4.2 ± 0.8, after: 5.6 ± 0.8, t = 6.425, *p* < 0.001, Cohen’s d = 1.785). The effort/importance was significantly higher after the course (median 7.0, IQR 6.3–7.0) than before the course (median 6.3, IQR 5.3–6.7) (W = 2.5, Z = 2.863, *p* = 0.004, r = 0.640). Self-efficacy increased significantly from before compared to after the BLS course (3.6 ± 2.0, and 7.4 ± 1.7, respectively; t = 9.794, *p* < 0.001, Cohen’s d = 2.111).

**Figure 3:**
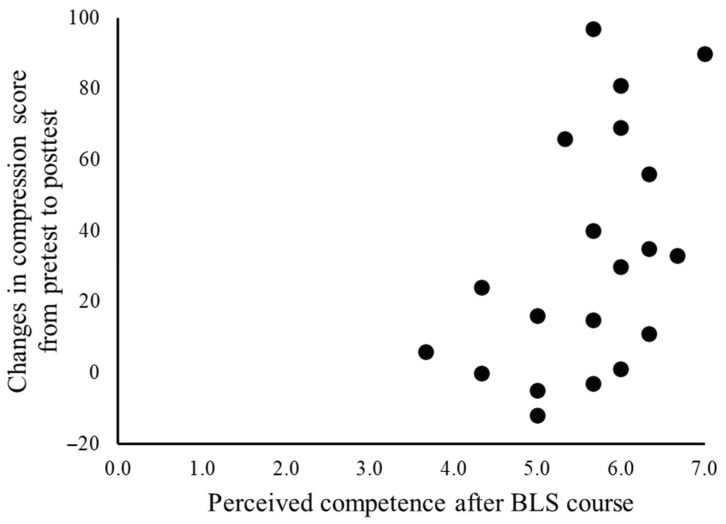
Correlation between compression score changes from pretest to posttest and perceived competence score after basic life support (BLS) training. Changes in the chest compression score between the pretest to the posttest were regarded as the effect of the BLS course. Perceived competence is a sub-scale of intrinsic motivation inventory (Min: 0.0, Max: 7.0) (r = 0.483, *p* = 0.031).

Although there was no significant correlation between the extent of change in the chest compression score and perceived competence before training (r = 0.075, *p* = 0.755), a significant correlation with perceived competence after training was found (r = 0.483, *p* = 0.031) (Figure 3). There was no significant correlation between the extent of change in the chest compression score and interest/enjoyment (before training: r = 0.389, *p* = 0.090, after training: r=0.274, *p* = 0.243) or effort/importance (before training: r = 0.177, *p* = 0.456, after training: r=0.268, *p* = 0.253). No significant correlation was found between the extent of change in the chest compression score and self-efficacy score (before training: r = 0.209, *p* = 0.377, after training: r = 0.304, *p* = 0.193).

## 4. Discussion

In the present study, we measured CPR skills quantitatively, and intrinsic motivation and self-efficacy before and after a CPR training program, to clarify the effect of CPR training and the relationship between the psychological aspects and CPR skill acquisition. The intrinsic motivation was scored with interest/enjoyment, perceived competence, and effort/importance sub-scales of the IMI. To the best of our knowledge, this is the first study investigating the relationship between the acquisition of CPR skill and intrinsic motivation, which is one of the psychological variables of a trainee and a factor that affects motor learning.

Similar to previous studies, the results of the present study showed that CPR training improved the compression score, which is the overall score for chest compressions [15,37]. However, the change induced by training was confirmed for each parameter; although compression depth showed significant improvement, the percentage of compressions fully released was found to be significantly worsened. In addition, the results of this study showed that CPR training improved the trainees’ intrinsic motivation and self-efficacy along with CPR skills. Partiprajak and Thongpo also reported that CPR training enhanced trainees’ self-efficacy [38]. Moreover, a significant association between self-efficacy to CPR at the community level and provision of bystander CPR and survival to discharge in OHCA patients have been reported [39]. Therefore, our results suggest that CPR training facilitates a desirable state in improving not only CPR skills but also psychologically, including self-efficacy. Although the correlation coefficient between self-efficacy and CPR skills was comparable with those of previous studies [29,30], we could not detect a significant association because our sample size was smaller than that reported in other studies. However, our study clarified that perceived competence after training has a significant correlation with the amount of CPR skills acquired by training. This result suggests that perceived competence after training has a stronger association with the change in CPR skills acquired through training than does self-efficacy and CPR skills. Therefore, CPR skills can be effectively improved by enhancing the perceived competence of trainees during the training. In the motor learning research domain, Abbas and North [34] examined the effect of feedback after good and poor performance on learners’ self-efficacy and intrinsic motivation using a golf putting task. Their results indicated that the group that received feedback on good performance showed more accurate performance, higher self-efficacy, and intrinsic motivation in the one-week retention test than did the group that received feedback on poor performance. Thus, it has been considered that the state of intrinsic motivation of learners during skill acquisition affects motor learning effectiveness [21]. Because the process of acquiring CPR skills is related to motor learning, we consider that the acquisition of these skills can also be promoted by increasing the intrinsic motivation of trainees in CPR training. However, the relationship between perceived competence after training and the change in CPR skill must be interpreted carefully, because various factors are involved in the acquisition of motor skills in a complex manner [40]. Perceived competence is not the only factor that determines the effectiveness of training. This fact is also evidenced by the result that the correlation between ability and learning effect was only moderate.

Moreover, our results also suggest the limits feedback provided by instructors may have. Studies comparing conventional training by instructors and training using a feedback device reported that training using a feedback device was more effective than conventional training [12,13,36,41]. Spooner et al. found the same results as above; however, their results showed this effectiveness not only immediately after training, but also after 6 weeks of training [41]. In addition, Cortegiani et al. compared the group trained with feedback from the instructors only and the group trained with feedback from the instructor and feedback devices and reported that the effects of training were significantly higher in the group that additionally used the feedback device than in the group that provided feedback from instructors only [13]. Cortegiani et al. reported that the median percentage of compressions fully released was 71% in the feedback device group and 24% in the conventional group. They also argued that the chest recoil may be difficult to teach and may not be recognized during chest compression performance without the use of feedback devices [13]. Moreover, it has been reported that the assessment from instructors on trainees’ chest compressions, especially the rate of chest compressions, was inaccurate. Brennan et al. verified the accuracy of the instructor’s assessment by comparing the measurements obtained from the simulation mannequin with the instructor’s measurements [42]. The result clarified that the instructor tends to judge the rate of chest compressions exceeding the range indicated by the guideline as "adequate", while judging the rate of chest compressions within the appropriate range as “inadequate”. Trainees are expected to correct their errors based on feedback to improve their CPR skills. Therefore, if they are not given accurate feedback, their error will not be corrected. Thus, while feedback plays an important role in training, there is a limit to how instructors can detect trainees’ errors and provide appropriate feedback because the instructor’s assessment of trainees’ CPR skills is less accurate than a device-based assessment. In this study, CPR training did not improve the rate of chest compressions; therefore, we assumed that there was a problem with the feedback provided by the instructor to the trainees during the training. In this respect, the feedback device can detect the error of the trainees’ performance in real time, quantitatively, and enhance the training effect.

This study had some limitations. First, this study was conducted during the coronavirus disease pandemic; thus, it was necessary to exclude ventilation from the measurement, to not increase the risk of infection. Therefore, it was not possible to investigate the effect of CPR training on ventilation-related indices and the relationship between changes in ventilation skills and psychological variables. Second, this study adopted a traditional training method based on instructor guidance without the use of feedback devices. This was because mannequins equipped with feedback devices in BLS training remain expensive, and traditional training remains universal. However, while training with instructors, the means and types of feedback may differ with each instructor. Since the way feedback is provided affects skill acquisition, intrinsic motivation, and self-efficacy, it is possible that the instructor’s involvement may have affected the results of this study. Finally, this was a quasi-experimental study to investigate the relationship between CPR skill acquisition and a psychological variable of trainees. Therefore, the present study was not meant to be an experimental design to identify causality. A randomized controlled trial is necessary to verify whether training that enhances perceived competency is truly effective for the acquisition of CPR skills in the future.

## 5. Conclusions

Our study revealed that CPR training improved trainees’ CPR skills, intrinsic motivation, and self-efficacy. We found a significant correlation between trainees’ perceived competency and the short-term learning effects of CPR training. This result suggests that CPR training organized to increase trainees’ perceived competency may prompt the acquisition of CPR skills. In the future, it is necessary to conduct randomized controlled trials to clarify whether training, which is conducted to be able to boost trainee’s competence, enhances the effectiveness of CPR training.

## Data Availability

All data generated or analyzed for the purposes of this study are available from the corresponding author upon request.
